# Muscle fiber characteristics and expression level of *Troponin T3*, *Toll-like receptor 2*, and *Toll-like receptor 4* genes in chicken meat with white striping

**DOI:** 10.14202/vetworld.2023.1415-1420

**Published:** 2023-07-09

**Authors:** Antika Boonlaos, Muhammad Jasim Uddin, Katchaporn Temyord, Danai Jattawa, Autchara Kayan

**Affiliations:** 1 Department of Animal Science, Faculty of Agriculture, Kasetsart University, Bangkok, Thailand; 2 School of Veterinary Medicine, Murdoch University, Western Australia, Australia; 3 Center for Biosecurity and One Health, Harry Butler Institute, Murdoch University, Murdoch, Australia; 4 Bureau of Livestock Standard and Certification, Department of Livestock Development, Bangkok, Thailand

**Keywords:** chicken, gene expression, myopathy, poultry, white striping

## Abstract

**Background and Aim:**

The poultry industry faces an emerging muscular defect in chicken meat called white striping (WS). The biological processes associated with WS myopathy are immune system activation, angiogenesis, hypoxia, cell death, and striated muscle contraction. We examined the *Troponin T3* (*TNNT3*), *Toll-like receptor 2* (*TLR2*), and *Toll-like receptor 4* (*TLR4*) genes based on their functions related to muscle contraction and the innate immune system. This study aimed to determine the muscle fiber characteristics (MFCs) and expression level of *TNNT3*, *TLR2*, and *TLR4* genes in white striping chicken meat (WSCM).

**Materials and Methods:**

A total of 428 breast samples were randomly collected from a commercial poultry processing plant. The samples were classified into four levels: 0 (normal), 1 (moderate WS), 2 (severe WS), and 3 (extreme WS). Five samples per group were selected to evaluate MFCs, including total number of muscle fibers, muscle fiber diameter, cross-sectional area, endomysium thickness, and perimysium thickness. Five samples per group were selected for ribonucleic acid (RNA) isolation to evaluate the messenger RNA (mRNA) expression levels of *TNNT3*, *TLR2*, and *TLR4* genes related to WS.

**Results:**

Statistical analysis revealed that the total number of fibers, endomysium thickness, and perimysium thickness significantly differed between groups (p < 0.05). Muscle fiber diameter and cross-sectional area did not significantly differ (p > 0.05). The expression of the *TNNT3* gene did not significantly differ among groups (p > 0.05). *Toll-like receptor 2* and *TLR4* mRNA expression significantly differed among groups (p < 0.05).

**Conclusion:**

These detailed MFCs will provide baseline information to observe WS in chicken meat. *Toll-like receptor 2* and *TLR4* genes may play a role in the occurrence of WS in chicken meat through non-specific immune reactions.

## Introduction

Chicken is the most-consumed meat in the world and a vital source of protein worldwide. Modern poultry industries are rearing fast-growing chickens to keep up with demand. The growth performance of chickens depends on many factors, including breeding, feeding, preventive, and management systems. The poultry industry faces an emerging muscular defect in chicken meat known as white striping (WS). White striping is an abnormal feature consisting of inserting white lines of fat and connective tissues along with normal muscle fibers. It negatively affects the appearance of meat and qualities such as tenderness, protein content, and consumer acceptability. White striping negatively impacts meat quality due to the deposition of fat in the muscle fiber because consumers prefer chicken breast meat with high protein and low fat. Consequently, consumer rejection has increased [1]. In addition, the thawing and cooking loss of white striping chicken meat (WSCM) are higher than those of normal breast meat. White striping chicken meat is reported to have lower levels of proteins, including sarcoplasmic protein, as well as decreased marinade uptake and flavor retention. Due to its higher fat or connective tissue levels and lower protein content, water-holding capacity is decreased in WSCM [2, 3]. WS causes economic losses due to customer complaints about fillets affected by these myopathies [4–6]; thus, WS negatively impacts the poultry industry. Although its exact etiology has remained unknown, WS is thought to be genetic, caused by selective breeding to produce fast-growing commercial broiler lines. Histopathologically, WS occurs due to the degradation of fast-growing muscle fibers and rapid regeneration of the fibers with connective tissue or fat. Inflammatory cell infiltration at the lesion is commonly observed [7, 8]. The extent of WS varies, and WSCM can be classified into four levels based on the thickness of WS inserted into the muscle [7].

The most important biological processes associated with WS myopathy are immune system activation, angiogenesis, hypoxia, cell death, and striated muscle contraction [9]. Along with these biological processes, several candidate genes are reportedly involved in WS myopathy in broilers [10, 11]. *Troponin T3* (*TNNT3*), *Toll-like receptor 2* (*TLR2*), and *Toll-like receptor 4* (*TLR4*) genes were selected for the present study based on their functions. Troponin T (TnT) is a key calcium regulator of actin thin filament function and is required for striated muscle contraction. Three homologous genes have evolved in vertebrates to encode three muscle-type-specific TnT isoforms: *TNNT1* for slow skeletal muscle TnT, *TNNT2* for cardiac muscle TnT, and *TNNT3* for fast skeletal muscle TnT [12]. The composition of muscle fiber types may affect meat quality by influencing the content of postmortem metabolites in livestock [13]. *Troponin T3* has been identified as a potential candidate gene primarily involved in actin binding and myofibril composition [14]. A higher abundance of *TNNT3* has been found in myopathy of the *pectoralis major* muscle in fast-growing broilers, which could be caused by a reduced supply of oxygen to the breast muscle [15]. In addition, some self-proteins released by damaged cells are likely to act as stress or danger-associated molecular patterns in chronic inflammatory conditions [16]. Immunologically active molecules such as cytokines and chemokines have been linked to skeletal muscle injury recovery. Toll-like receptors (TLRs) serve as key sentinel molecules of the innate immune system in vertebrates [17]. Muscle atrophy is an active process regulated by specific transcriptional programs that result in the loss of muscle mass due to increased protein degradation and/or decreased protein synthesis. Toll-like receptors can activate an immune response in response to signals from damaged or stressed cells [16]. Therefore, TLRs play a key role in myopathies characterized by repeated bouts of muscle fiber degeneration and regeneration in an attempt to repair contraction-induced damage [18]. *Toll-like receptor 2* and *TLR4* appear to be particularly important in the early stages of inflammation activation in the case of myopathies [19]. *Toll-like receptor 2* and *TLR4* converge to increase the expression of proinflammatory cytokines in muscle cells [20].

However, there have been no reports on *TNNT3*, *TLR2*, or *TLR4* related to the occurrence of WS in chicken meat. Therefore, this study aimed to determine the muscle fiber characteristics (MFCs) and *TNNT3*, *TLR2*, and *TLR4* gene expression levels in WSCM.

## Materials and Methods

### Ethical approval

This study analyzed that the meat samples were taken from a commercial poultry processing plant, which did not need contact with animals. This study was not involved any invasive procedure on animal; therefore, ethical approval is not required.

### Study period and location

This study was conducted from June 2020 to December 2022. All muscle samples were randomly selected from a commercial poultry processing plant in Pathum Thani, Thailand. The Department of Animal Science, Faculty of Agriculture, Kasetsart University processed the samples for further analysis.

### Experimental animals

Breast (*pectoralis major*) muscle samples were randomly selected from a total of 428 broilers processed in a commercial poultry processing plant. All chickens were slaughtered on 42 days of age and had live weights of 2.5–3 kg according to standard slaughtering procedures of the Department of Livestock Development, Thailand. The breast muscles were classified into four levels of WS: level 0 (normal), no white line intramuscularly; level 1 (moderate WS), WS insertion thickness <1 mm; level 2 (severe WS), thickness of WS insertion 1–2 mm; and level 3 (extreme WS), thickness of WS inserted in the muscle >2 mm [1]. The muscle samples were collected, and superficial fat, cartilage, and connective tissues were trimmed. The samples were then kept at −80°C for further analysis.

### Histochemical analysis

Breast (*pectoralis major*) muscle samples were used for the histological studies. Twenty meat samples were selected (five samples per group) from a total of 428 broiler samples. The samples were cut into 0.5 × 0.5 × 1.0 cm pieces and immediately fixed in 10% buffered neutral formalin solution for 24 h. The samples were then dehydrated in alcohol, cleared in xylene, infiltrated, and embedded in paraffin. The sections were cut to 3 μm thickness and stained with hematoxylin and eosin for general histological study. Stained cross-sections were photographed using a light microscope (OlympusFSX100, Olympus, Tokyo, Japan) with a 10× objective lens and a 10× eyepiece. Five photographs of different cross-sections from each muscle were taken. The samples were analyzed using Image-J software (National Institute of Mental Health, Bethesda, MD, USA). The mean number of fibers per area was obtained by counting the total number of fibers (TNF) in five areas (582,007 μm^2^ each) per sample. The mean of approximately 300 fibers in five random fields for each muscle was measured to estimate the fiber diameters (μm) and fiber areas (μm^2^). The thicknesses of the endomysium and perimysium were determined for each sample. The structural elements were measured in the area of the fiber bundle. Forty measurements of the thickness (μm) of endomysium and ten measurements of the thickness (μm) of perimysium were taken on each image. The mean thickness was estimated from the measured values [21].

### Messenger ribonucleic acid (mRNA) expression of *TNNT3, TLR2*, and *TLR4* genes

Total RNA was isolated from each breast (*pectoralis major*) muscle sample (n = 5 per group) using a QIAamp RNA Mini Kit (Qiagen, Courtaboeuf, France) according to the manufacturer’s recommendations. The purity of the extracted RNA was measured using a NanoDrop spectrophotometer. Real-time polymerase chain reaction (PCR) analysis was performed on a MyGo Pro® real-time PCR instrument (IT-IS Life Science Ltd., Middlesbrough, UK) with a reaction mixture using a QuantiNova SYBR green reverse-transcription PCR (RT-PCR) Kit (Qiagen, Hilden, Germany) consisting of 10 μL of 2× QuantiNova SYBR Green RT-PCR Master Mix, 1 μL each of 10 μM (0.5 μM) forward and reverse primer, 0.2 μL of QN SYBR Green RT Mix, 5 μL of template, and 2.8 μL of nuclease-free water added for a total reaction volume of 20 μL. A two-step amplification program was performed pre-denaturation at 95°C for 2 min, followed by 40 cycles of denaturation at 95°C for 5 s and annealing and extension at 60°C for 10 s. All samples were repeated twice per sample, and the mean of the two replications was used. Final results were reported as the relative expression level after normalization of the transcript level using the housekeeping gene *ß-actin*. Polymerase chain reaction primers were designed using Primer3 software (https://primer3.ut.ee) (Table-1) [22, 23].

**Table-1:** Quantitative reverse-transcription PCR primer sequences.

Name	Sequence	Tm (°C)	Reference
*TNNT3*			
Forward	*5’*-ACCCCACTCCCTTCCTTTC-*3’*	60	-
Reverse	*5’*-TTGGGGGTGTGGAGACAG-*3’*		
*TLR2*			
Forward	*5’*-CTGATCCTGTGCCAATCAGA-*3’*	60	[23]
Reverse	*5’*-CCTGGTGCTCCATCTCAAGT-*3’*		
*TLR4*			
Forward	*5’*-ACAGGTGCCACATCCATACA-*3’*	60	[23]
Reverse	*5’*-TATGGCCCAGATTCAGCTCC-*3’*		
*ß-actin*			
Forward	*5’*-CCACCGCAAATGCTTCTA-*3’*	60	[23]
Reverse	*5’*-GCCAATCTCGTCTTGTTTTATG-*3’*		

PCR=Polymerase chain reaction, *TNNT3=Troponin T3, TLR2=Toll-like receptor 2, TLR4=Toll-like receptor 4*

### Statistical analysis

The differences between groups were analyzed by analysis of variance using SAS version 9.2 (SAS Inst. Inc., Cary, NC, USA). Values of p < 0.05 were considered to indicate statistically significant differences between groups. Significant differences (p < 0.05) among means were identified using Proc GLM procedures. The results are presented as least squares means with standard errors.

The model used in statistics was:

γ_ij_ = μ + τ_i_ + ε_ij_ (1)

γ_ij_ = is the observation of the trait, τ_i_ = effect of treatment (i = white striping level 0, 1, 2, and 3), ε_ij_ = the random residual error.

## Results

### Muscle fiber characteristics

Muscle fiber characteristics were examined in breast samples with levels 0, 1, 2, and 3 WS. Histopathological study showed muscular dystrophy at higher percentages with increasingly severe WS. The muscle fibers were surrounded by fibrous or connective tissue. A thicker endomysium and perimysium were also observed in samples with higher levels of WS. The muscle fiber diameter and cross-sectional area did not significantly differ between groups (p > 0.05). The TNF for levels 0 and 1 was higher (p < 0.01) than that of level 3 WSCM (Table-2). Level 3 WSCM showed the greatest endomysium thickness (p < 0.001). Perimysium thickness was higher in levels 2 and 3 WSCM (p < 0.001) than in levels 0 and 1.

**Table-2:** Muscle fiber characteristics in different levels of white striping (mean ± standard error).

Parameters	Level of white striping				p-value

	0	1	2	3	
TNF	293.33 ± 3.92^a^	254.13 ± 15.55^a^	245.67 ± 16.11^ab^	201.74 ± 14.15^b^	0.002
MFD (μm)	69.24 ± 1.49	65.15 ± 1.27	61.53 ± 3.15	69.13 ± 2.24	0.068
CSA (μm^2^)	3,752.09 ± 117.90	3,994.55 ± 214.42	3,642.81 ± 185.43	4,160.92 ± 253.95	0.284
ET (μm)	6.68 ± 0.07^a^	8.72 ± 0.15^b^	9.76 ± 0.18^c^	11.30 ± 4.99^d^	<0.001
PT (μm)	13.97 ± 0.53^a^	16.96 ± 0.56^b^	23.78 ± 0.66^c^	25.99 ± 0.10^c^	<0.001

TNF=Total number of fibers, MDF=Muscle fiber diameter (μm), CSA=Cross-section area (μm^2^), ET=Endomysium thickness, PT=Perimysium thickness (μm). ^a-d^Means with different superscripts in the same row indicate a significant difference (p < 0.05)

### Messenger ribonucleic acid expression of *TNNT3, TLR2*, and *TLR4* genes

**Figure-1:**
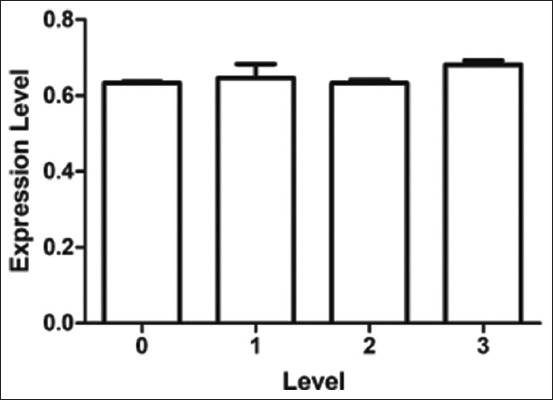
Expression level of *Troponin T3* gene transcript in difference levels of white striping. White striping (WS) levels were Level 0 (normal) = 0, Level 1 (moderate WS) = 1, Level 2 (severe WS) = 2, Level 3 (extreme WS) = 3.

**Figure-2:**
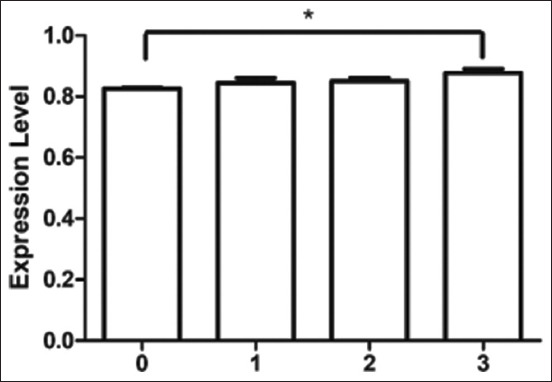
Expression level of *Toll-like receptor 2* gene transcript at different levels of white striping. White striping (WS) levels were Level 0 (normal) = 0, Level 1 (moderate WS) = 1, Level 2 (severe WS) = 2, Level 3 (extreme WS) = 3.

**Figure-3:**
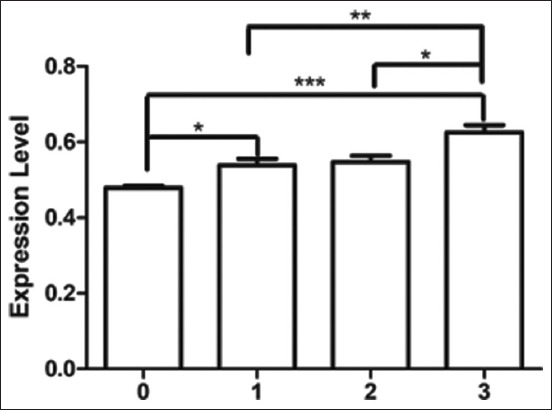
Expression level of *Toll-like receptor 4* gene transcript at different levels of white striping. White striping (WS) levels were Level 0 (normal) = 0, Level 1 (moderate WS) = 1, Level 2 (severe WS) = 2, Level 3 (extreme WS) = 3.

Quantitative real-time PCR showed the abundance of *TNNT3* transcripts at different levels of WS. *Troponin T3* mRNA expression did not significantly (p > 0.05) differ from WS level (Figure-1). The abundance of *TNNT3* transcripts at WS levels 0, 1, 2, and 3 was 0.633 ± 0.004, 0.646 ± 0.037, 0.633 ± 0.008, and 0.681 ± 0.012, respectively. The mRNA expression abundance of *TLR2* was significantly higher (p < 0.05) in extreme (level 3) WSCM compared to normal chicken meat (Figure-2). The abundance of *TLR2* transcripts at WS levels 0, 1, 2, and 3 was 0.825 ± 0.004, 0.845 ± 0.015, 0.851 ± 0.011, and 0.877 ± 0.013, respectively. The mRNA expression of *TLR4* was significantly different among groups (p < 0.001) (Figure-3), except between levels 1 and 2. The abundance of *TLR4* transcripts at WS levels 0, 1, 2, and 3 was 0.479 ± 0.005, 0.538 ± 0.017, 0.547 ± 0.017, and 0.625 ± 0.020, respectively.

## Discussion

Histological analysis showed a greater total number of muscle fibers, endomysium thickness, and perimysium thickness in chicken meat with higher levels of WS. In WS, the breast muscles are damaged by multifocal muscular degeneration and necrosis at various phases of fast development, which is linked with muscle regeneration, fibrosis, and adipose tissue infiltration. The severity of the muscle lesions increases from normal breasts (score 0) to breasts with severe lesions (score 2) [6]. Histological analysis has shown that during the occurrence of WS, there is muscle fiber degeneration and regeneration with fibrosis and lipidosis, hyaline degeneration, inflammatory cell infiltration, multifocal edema, and nuclear internalization. Consequently, the muscle fibers are replaced by connective tissue and lipids [7, 8]. Myopathies feature overlapping lesions, including polyphasic myodegeneration, perivascular inflammatory cuffing, and fibrous tissue and fat accumulation [24]. Muscle fibrosis is replaced by extracellular matrix proteins such as collagen in the muscle fibers. The structure of collagen fibrils in connective tissue sections around muscle fiber bundles (perimysium) and around individual muscle fibers is connected to the tissue’s ability to flex and stretch (endomysium) [25]. Muscle fiber degradation begins after 2 weeks of growth and increases rapidly within 28 days. On 46 days, there is a lack of normal cross-striations, a huge necrotic process, degenerating fibers infiltrated by inflammatory cells, scattered fibers in an abundant collagen-rich connective tissue, and a high percentage of apoptotic nuclei in necrotic fibers [26]. Broiler genetic selection for fast growth may have driven muscle fibers to their maximum functional size restrictions [27].

We did not find a significant difference in *TNNT3* mRNA expression among the different levels of WS in chicken breast muscle. However, there was a trend of numerically higher *TNNT3* gene expression with extreme WSCM. A previous study reported a higher abundance of the *TNNT3* gene in myopathy of the *pectoralis major* in fast-growing broilers [15]. The mRNA expression of *TLR2* and *TLR4* significantly differed among WS groups. In general, mRNA expression was higher in muscles with a higher level of WS. The most relevant biological processes identified in WS myopathy include immune system activation, angiogenesis, hypoxia, cell death, and striated muscle contraction [28]. Apoptosis in muscle cells affected by myopathy causes tissue damage that activates the repair process. Several genes associated with the immune system are involved in the repair mechanism [9]. Toll-like receptors activate a non-specific immune mechanism in response to signals from damaged or stressed cells [16]. Toll-like receptors are involved in muscular dystrophies by repeated bouts of muscle fiber degeneration and regeneration in an attempt to repair contraction-induced damage [18]. *Toll-like receptor 2* and *TLR4* are particularly important at the early stages of inflammation activation [19]. *Toll-like receptor 4* and *TLR2* converge to increase the expression of proinflammatory cytokines in muscle cells, which causes mild inflammation, connective tissue formation, and fat deposition in damaged tissues [20]. Toll-like receptor-mediated signaling is critical in regulating macrophage and inflammatory cell infiltration during regenerative myogenesis in response to acute and chronic muscle injury. *Toll-like receptor 2* and *TLR4* signaling are required for skeletal muscle repair after an acute injury [29]. Histological evidence indicates that WS-affected breast muscle has abnormal fiber development with a gross lesion of compromising muscle composition [25]. Many metabolic processes are shared by WS, including interstitial inflammatory infiltration, fibrosis, and altered cell regeneration [3]. The findings of the present study indicate the involvement of *TLR2* and *TLR4* genes in the occurrence of WS in chicken meat.

## Conclusion

A greater TNF, endomysium thickness, and perimysium thickness were found with higher levels of WS in chicken meat. *Toll-like receptor 2* and *TLR4* mRNA expression levels in chicken meat varied at different levels of WS. A higher expression of *TLR2* and *TLR4* genes was detected in meat at the highest level of WS, which indicates that these genes might be involved in WS. However, further functional studies are warranted to investigate the mechanism of WS in breast meat.

## Authors’ Contributions

AK and AB: Conducted the study, analyzed data, and drafted and revised the manuscript. MJU, KT, and DJ: Provided technical help during the experiments. AK and MJU: Study conception and design and reviewed the manuscript. All authors have read, reviewed, and approved the final manuscript.
